# ﻿Morpho-phylogenetic evidence reveals *Pseudolomaanthathailandica* gen. et sp. nov. and *Submultiguttulisporamultiseptata* gen. et sp. nov. in Chaetosphaeriaceae

**DOI:** 10.3897/mycokeys.113.142643

**Published:** 2025-01-31

**Authors:** Jing-Yi Zhang, Kevin D. Hyde, Jian Ma, Na Wu, Fatimah Al-Otibi, Li-Juan Zhang, Yong-Zhong Lu

**Affiliations:** 1 School of Food and Pharmaceutical Engineering, Guizhou Institute of Technology, Guiyang 550003, China; 2 Center of Excellence in Fungal Research, Mae Fah Luang University, Chiang Rai 57100, Thailand; 3 School of Science, Mae Fah Luang University, Chiang Rai 57100, Thailand; 4 Department of Botany and Microbiology, College of Science, King Saud University, P.O. Box 22452, Riyadh 11495, Saudi Arabia; 5 Guizhou Industry Polytechnic College, Guiyang 550008, China; 6 School of Life Science and Technology, Center for Informational Biology, University of Electronic Science and Technology of China, Chengdu 611731, China

**Keywords:** 4 new taxa, asexual morph, Sordariomycetes, sporidesmium-like fungus, taxonomy

## Abstract

Anamorphic chaetosphaeriaceous fungi exhibit high morphological variability and are distributed worldwide across terrestrial and aquatic habitats. During an ongoing taxonomic study of microfungi, two intriguing chaetosphaeriaceous hyphomycetes were collected from dead wood and dead bamboo stems in China and Thailand. A polyphasic approach, combining morphological characteristics and phylogenetic analysis of LSU and ITS sequence data, revealed that these fungi represent two new genera within Chaetosphaeriaceae. *Pseudolomaantha* and *Submultiguttulispora* are proposed for these new genera, and they exhibit non-phialidic and phialidic asexual morphs, respectively. *Pseudolomaanthathailandica***gen. et sp. nov.** is characterized by a sporidesmium-like asexual morph with macronematous, mononematous conidiophores; monoblastic conidiogenous cells, and pyriform to obclavate, rostrate conidia bearing an apical appendage. *Submultiguttulisporamultiseptata***gen. et sp. nov.** is distinguished by macronematous, mononematous conidiophores, mono- to polyphialidic conidiogenous cells, and fusiform or ellipsoidal-fusiform, pale brown to olive green to brown conidia with filiform, hyaline appendages at both ends. Detailed descriptions, illustrations, and notes on the new collections are provided, along with a key to non-phialidic hyphomycetous genera in Chaetosphaeriaceae.

## ﻿Introduction

Chaetosphaeriales was established by Huhndorf et al. (2004) to accommodate the family Chaetosphaeriaceae based on morphological characteristics and phylogenetic analysis of LSU sequence data. Currently, four families, *viz.*, Chaetosphaeriaceae, Helminthosphaeriaceae, Leptosporellaceae, and Linocarpaceae are recognized within this order (Hyde et al. 2020; Wijayawardene et al. 2022). The estimated stem age of Chaetosphaeriales is approximately 158 MYA, based on divergence time analysis (Hyde et al. 2020).

Chaetosphaeriaceae was invalidly introduced by Locquin (1984) without a formal description but was later validly re-established by Réblová et al. (1999) to accommodate *Chaetosphaeria* and its relatives. Since its re-establishment, the family has shown remarkable diversity with a significant increase in the number of genera and species described in recent years (Lin et al. 2019; Yang et al. 2019; Réblová et al. 2020, 2021a, b, c, d, e, 2022, 2024; Wijayawardene et al. 2022; Wu and Diao 2022, 2023; Réblová and Nekvindová 2023; Yang et al. 2023; Hyde et al. 2024a). Wu and Diao (2022) conducted a comprehensive study of the anamorphic Chaetosphaeriaceae, which included 89 genera, establishing the family as one of the largest within Sordariomycetes (Wijayawardene et al. 2022). Their study also provided identification keys for most genera (Wu and Diao 2022). Following this, Réblová et al. (2022) and Réblová and Nekvindová (2023) carried out systematic reviews of chloridium-like morphotypes, resulting in the addition of seven new genera to Chaetosphaeriaceae. In subsequent years, five more new genera, *viz.*, *Gongromerizella*, *Neocirrenalia*, *Paragongromeriza*, *Pseudophialocephala* and *Pseudostriatosphaeria*, were introduced based on morphology and phylogeny (Manawasinghe et al. 2022; Yang et al. 2023; Wu and Diao 2023; Tian et al. 2024; Zhang et al. 2024). Réblová et al. (2024) re-evaluated species in genera *Exserticlava*, *Phaeostalagmus*, *Phialocephala*, and several chalara- and stanjehughesia-like fungi, which led to the establishment of three new genera in Chaetosphaeriaceae. On the other hand, *Ellisembia* was removed from Chaetosphaeriaceae and reclassified under Sporidesmiaceae (Delgado et al. 2024; Hyde et al. 2024a). Hyde et al. (2024a) accepted 107 genera in Chaetosphaeriaceae.

The sexual morph of Chaetosphaeriaceae is characterized by perithecial, papillate, globose to subglobose, setose, dark brown to black ascomata; unitunicate, clavate to cylindrical asci with a J-, apical ring; and 0–3-septate, fusiform, cylindrical to ellipsoid, hyaline to brown ascospores, often with guttules, a sheath, or appendages (Réblová et al. 1999; Réblová and Gams 2000; Hyde et al. 2020; Wu and Diao 2022). The asexual morphs of Chaetosphaeriaceae include both hyphomycetes and coelomycetes. Coelomycetous morphs are characterized by stromatic, cupuliform or globose, unilocular, setose conidiomata; numerous, septate, ovoid to cylindrical setae; 4–6-septate, unbranched, pigmented conidiophores; integrated, holoblastic or enteroblastic, phialidic conidiogenous cells with conspicuous periclinal thickening at an attenuated apex; and aseptate, hyaline to brown conidia with tubular appendages at the ends (Hashimoto et al. 2015; Hyde et al. 2020; Li et al. 2020). Hyphomycetous morphs are further divided into phialidic and non-phialidic anamorphs. Phialidic anamorphs exhibit macronematous, mononematous, septate, pigmented conidiophores; mono- or polyphialidic conidiogenous cells that proliferate percurrently or sympodially, often with funnel-shaped collarettes; and aggregated, fusiform, allantoid, cylindrical or doliiform conidia, which are mostly hyaline but sometimes pigmented, and often possess filiform appendages (Réblová 2004; Fernández and Huhndorf 2005; Liu et al. 2016; Lin et al. 2019; Luo et al. 2019; Yang et al. 2019; Réblová et al. 2020, 2021a, b, d). Non-phialidic fungi in Chaetosphaeriaceae are predominantly characterized by sporidesmium-like asexual morphs (Ellis 1971, 1976; Wu and Zhuang 2005; Wu and Diao 2022; Yang et al. 2023; Delgado et al. 2024). These taxa are primarily saprobic, occurring on various plant substrates in both terrestrial and aquatic habitats, with some species also found in soil or as fungicolous taxa (Hughes and Kendrick 1968; Perera et al. 2016; Hyde et al. 2018; Réblová et al. 2020, 2021d; Wu and Diao 2022; Zhang et al. 2022; Calabon et al. 2023; Yang et al. 2024; Zhang et al. 2024).

In this study, we aim to introduce two new genera, *Pseudolomaantha* and *Submultiguttulispora*, to accommodate two new species, *P.thailandica* and *S.multiseptata*, respectively. Evidence from morphology and phylogenetic analysis of a combined LSU and ITS sequence dataset supports the establishment of these two new genera (*Pseudolomaantha* and *Submultiguttulispora*) within Chaetosphaeriaceae, Chaetosphaeriales, Sordariomycetes.

## ﻿Material and methods

### ﻿Collections, isolation and conservation

Samples of dead bamboo stems and wood were collected from Thailand and China. The collection information of the samples was noted (Rathnayaka et al. 2024), and the samples were taken to the laboratory in zip-lock plastic bags and subsequently examined using the methods described in Senanayake et al. (2020). Morphological observations of the fungal colonies on natural substrates were conducted using a stereomicroscope (Leica EZ4 Microsystems (Schweiz) AG, Singapore). A detailed examination of fungal structures was carried out using a Nikon ECLIPSE Ni compound microscope (Nikon, Japan) and photographed with a Nikon DS-Ri2 digital camera attached to the microscope. Measurements of fungal structures were made using Tarosoft^®^ Image Frame Work, and images used in figures were processed and assembled with Adobe Illustrator CS6 (Adobe Systems, San Jose, CA, USA).

Single-spore isolations were performed on water agar (WA), and germinated spores were transferred to potato dextrose agar (PDA) to obtain pure cultures (Chomnunti et al. 2014). Dried specimens were deposited in the Herbarium of Mae Fah Luang University (MFLU), Chiang Rai, Thailand, and the Herbarium of Cryptogams, Kunming Institute of Botany, Academia Sinica (HKAS), Kunming, China, and the Herbarium of Guizhou Academy of Agricultural Sciences (GZAAS), Guiyang, China. Pure cultures were deposited in the Mae Fah Luang University Culture Collection (MFLUCC) and the Kunming Institute of Botany Culture Collection (KUNCC). Faces of Fungi and Index Fungorum numbers were registered following the guidelines of Jayasiri et al. (2015) and Index Fungorum (http://www.indexfungorum.org/Names/Names.asp; accessed on 15 November 2024)

### ﻿DNA extraction, PCR amplification and sequencing

Pure cultures were incubated at 25 °C–28 °C for one month. Fresh fungal mycelia were scraped from the surface of the colonies and transferred to a 1.5 mL microcentrifuge tube using a sterilized scalpel for genomic DNA extraction. Genomic DNA was extracted using the Biospin Fungus Genomic DNA Extraction Kit (Biospin Fungus Genomic DNA Extraction Kit, BioFlux®, Shanghai, China) following the manufacturer’s instructions. The large subunit of ribosomal DNA (LSU) and the internal transcribed spacer (ITS) gene regions were amplified using primers LR0R and LR5 (Vilgalys and Hester 1990) and ITS5 and ITS4 (White et al. 1990), respectively. Polymerase chain reaction (PCR) was performed in a 50 µL reaction mixture containing 2 µL of DNA template, 2 µL of each forward and reverse primer (10 µM), 25 µL of 2 × Taq PCR Master Mix with blue dye (Sangon Biotech, China), and 19 µL of distilled–deionized water. Amplification conditions for the LSU and ITS regions followed the protocol described by Zhang et al. (2022). The quality of PCR products was assessed using 1% agarose gel electrophoresis stained with ethidium bromide. Purification and sequencing of PCR products were performed by Beijing Qingke Biotechnology Co., Ltd.

### ﻿Phylogenetic analyses

Original sequences were verified using BioEdit v. 7.1.3.0 (Hall 1999), and were assembled using SeqMan v. 7.0.0 (DNASTAR, Madison, WI, USA). The newly generated sequences were subjected to BLAST searches in GenBank to determine closely related taxa. Taxa used in the phylogenetic analysis for Chaetosphaeriaceae were selected and obtained from previous studies and GenBank (Wu and Diao 2022; Zhang et al. 2022; Réblová and Nekvindová 2023; Réblová et al. 2024). Sequence alignments for each locus were aligned using the online multiple alignment program MAFFT v.7 (http://mafft.cbrc.jp/alignment/server/, accessed September 2024; Katoh et al. 2019). The alignments were visually checked and manually improved where necessary using BioEdit v. 7.1.3.0 (Hall 1999). LSU and ITS sequences were combined using SequenceMatrix 1.7.8 (Vaidya et al. 2011). Sequences generated in this study were deposited in GenBank (Table 1).

**Table 1.** Chaetosphaeriaceae taxa used in the phylogenetic analysis, and their corresponding GenBank accession numbers.

Taxon	Strain	Status	ITS	LSU
*Achrochaetarivulata*	CBS 148186		OR286508	OR286551
*Achrochaetatalbotii*	ICMP 15161		MT454480	MT454495
*Aciculadictyochaetaluquillensis*	SMH 2973		N/A	AF466074
*Adautomilaneziacaesalpiniae*	CC-LAMIC 102/12	T	KX821777	KU170671
*Anacacumisporiumappendiculatum*	HMAS 245593	T	KP347129	KT001553
*Anacraspedodidymumsubmerum*	YMF1.4176	T	MK165445	MK165443
*Arcuatosporanovae-zelandiae*	CBS 109474		MW984569	MW984552
*Arcuatosporaseorsa*	CBS 147510	T	MW984572	MW984555
*Aunstrupianodipes*	NN043149		OL627566	OL655011
*Brachydictyochaetaantillana*	NN058987		OL627951	OL655147
*Brachydictyochaetabulliformis*	NN076027		OL628023	OL655155
*Brunneodinemasporiumbrasiliense*	CBS 112007	T	JQ889272	JQ889288
*Brunneodinemasporiumjonesii*	GZCC 16–0050	T	KY026058	KY026055
*Cacumisporiumacutatum*	CBS 101312		AF178553	AF178553
*Cacumisporiumacutatum*	CBS 101315	T	OR134682	OR134626
*Cacumisporiumcapitulatum*	CBS 101313		OR134683	OR134627
*Caliciastrumbicolor*	ICMP 15136	T	OR134689	OR134633
*Caliciastrumbicolor*	PRA-21507	T	N/A	OR134634
*Caligosporadilabens*	CBS 734.83	T	OR134691	OR134636
*Caligosporadilabens*	CBS 735.83	T	MH861684	N/A
*Caligosporapannosa*	CBS 551.89	T	OR134692	OR134637
*Calvolachnellaguaviyunis*	CBS 134695	T	KJ834524	KJ834525
*Capillisphaeriacrustacea*	CBS 144665		OR134695	OR134640
*Capillisphaeriacrustacea*	ICMP 15139		OR134696	OR134641
*Catenulariaangulospora*	MFLUCC 18–1331		MK828638	MK835840
*Catenulariacatenulata*	DLUCC 0891	T	MK828637	MK835838
*Catenulariaminor*	PRM 900544	T	MW987827	MW987822
*Chaetosphaeriaguttulata*	MFLUCC 17–1703	T	MK828636	MK835837
*Chaetosphaeriainnumera*	M.R. 3775		OR134699	OR134644
*Chaetosphaeriainnumera*	CBS 145639		OP455358	OP455464
*Chaetosphaeriamangrovei*	MCD 069	T	MG813821	MG813820
*Chaetosphaeriapolygonalis*	GZCC 20–0438	T	OP377861	OP377946
*Chalarodesobpyramidata*	PDD 119364		MW987828	MW987823
*Chloridiumbellum*	CBS 709.73A	T	OP455360	OP455466
*Chloridiumcaesium*	CBS 145633		OP455367	OP455474
*Chloridiumgamsii*	CBS 667.75	T	OP455415	OP455522
*Chloridiumvirescens*	CBS 145481		OP455439	OP455547
*Codinaeaassamica*	CBS 139907	T	OL654077	OL654134
*Codinaeafertilis*	IMI 233824		OL654080	OL654137
*Codinaeapaniculata*	CBS 145098	T	MT118230	MT118201
*Codinaeellalambertiae*	CBS 143419	T	OL654084	OL654141
*Codinaeellaminuta*	CBS 280.59		OL654090	OL654147
*Codinaeellaparvilobata*	CBS 144536	T	OL654100	OL654157
*Conicomycespseudotransvaalensis*	HHUF 29956	T	LC001710	LC001708
*Craspedodidymumelatum*	NN042874		OL627547	OL655004
*Cryptophialeudagawae*	GZCC 18–0047		MN104608	MN104619
*Cryptophialoideafasciculata*	MFLU 18–1499		MH758195	MH758208
*Curvichaetacurvispora*	ICMP 15115	T	OR134705	OR134650
*Curvichaetacurvispora*	ICMP 15118		OR134706	OR134651
*Dendrophomacytisporoides*	CBS 144107		MT118234	MT118205
*Dictyochaetacallimorpha*	ICMP 15130		MT454483	MT454498
*Dictyochaetafuegiana*	ICMP 15153	T	MT454487	EF063574
*Dictyochaetaquerna*	CBS 145503		MT454489	MT454503
*Dinemasporiumcruciferum*	HHUF 30001		AB900895	AB934039
*Dinemasporiumpseudoindicum*	CBS 127402	T	JQ889277	JQ889293
*Ericiosphaeriaspinosa*	S.M.H. 2754	T	MW984575	AF466079
*Eucalyptostromaeucalypti*	CBS 142074	T	KY173408	KY173500
*Eucalyptostromahongluosiense*	NN076613		OL628127	OL655185
*Eucalyptostromiellabeijingensis*	NN078016		OL628501	OL655251
*Exserticlavavasiformis*	TAMA 450		N/A	AB753846
*Exserticlavopsischlorotunicata*	S.M.H. 1565	T	N/A	AF466064
*Falholtiakaohsiungensis*	NCYU108K3-1-1	T	MT939301	MT939304
*Falholtiakaohsiungensis*	NN050711		OL627699	OL655083
*Flectosporalaminata*	CBS 112964	T	MW984576	MW984558
*Fuscocatenulasubmersa*	MFLUCC 18–1342	T	MK828634	MK835835
*Fuscocatenulavariegata*	NN055332		OL627817	OL655124
*Fusichloridiumcylindrosporum*	CBS 101429	T	OR134709	OR134653
*Fusichloridiumcylindrosporum*	CBS 101430		OR134710	OR134654
*Geniculosetapreussii*	CBS 263.75		OR134713	OR134657
*Geniculosetapreussii*	CBS 145478		OR134714	OR134658
*Gongromerizamyriocarpa*	CBS 264.76		AF178552	AF178552
*Gongromerizamyriocarpa*	CBS 141.53	T	OP455456	OP455564
*Gongromerizapygmaea*	IMI 506815		OR134724	OR134668
*Gongromerizellapachytrachela*	CBS 645.75	T	OP455461	OP455569
*Gongromerizellapini*	CBS 146011	T	MT223787	MT223882
*Gongromerizellasilvana*	CBS 171.76	T	OR134729	OR134673
*Infundibulomycescupulatus*	BCC 11929	T	EF113976	EF113979
*Infundibulomycesoblongisporus*	BCC 13400	T	EF113977	EF113980
*Kionochaetamicrospora*	GZCC 18–0036	T	MN104607	MN104618
*Kionochaetaramifera*	MUCL 39164		MW144421	MW144404
*Kionochaetiellaivoriensis*	CBS 374.76	T	MH860988	MH872758
*Kylindrochaetalignomollis*	S.M.H. 3015	T	EU037896	AF466073
*Leptosporellaarengae*	MFLUCC 15–0330	T	MG272255	MG272246
*Leptosporellabambusae*	MFLUCC 12–0846	T	KU940134	KU863122
*Linkosiamultiseptum*	CGMCC 3.20786	T	OL627557	OL655008
*Linkosiarostrata*	CGMCC 3.20790	T	OL627662	OL655059
*Lomaanthaaquirostrata*	GZCC 20–0503	T	OP377802	OP377901
*Lomaanthaaurantiaca*	CBS 126743	T	HM241692	HM241692
*Lomaanthaaurea*	CBS 144403	T	MH836375	MH836376
*Lunatochaetashenzhenensis*	CGMCC 3.20757	T	OL628577	OL655258
*Menisporacaesia*	CBS 145022		OL654107	OL654164
*Menisporaciliata*	CBS 122131	T	EU488736	OL654165
*Menisporatortuosa*	CBS 117553		OL654111	OL654169
*Menisporopsispirozynskii*	MUCL 47217		MW984579	MW984561
*Menisporopsistheobromae*	MUCL 41079		MW984580	MW984562
*Morrisiellaindica*	NN042908		OL627551	OL655005
*Morrisiellaindica*	NN044710		OL627629	OL655037
*Multiguttulisporadimorpha*	CBS 140002		MW984582	MW984564
*Multiguttulisporatriseptata*	IMI 353690		MW984584	MW984566
*Nawawiafiliformis*	MFLUCC 17–2394		MH758196	MH758209
*Neonawawiamalaysiana*	CPC 16757	T	GU229886	GU229887
*Neopseudolachnellaacutispora*	MAFF 244358	T	AB934065	AB934041
*Neopseudolachnellamagnispora*	MAFF 244359	T	AB934066	AB934042
*Neocirrenalianigrospora*	MFLUCC 18–0418		OP377888	OP377974
*Nimesporellacapillacea*	IMI 358908	T	OL654114	OL654171
*Paliphoraintermedia*	CBS 896.97	I	MH862682	EF204501
*Papillosporahebetiseta*	CBS 102340	T	AF178549	AF178549
*Paraceratocladiellapolysetosa*	NN044119		OL627605	OL655027
*Paraceratocladiumsilvestre*	NN055375		OL627830	OL655132
*Paracryptophialepirozynskii*	CGMCC 3.20706	T	OL627641	OL655047
*Paragaeumannomycespanamensis*	S.M.H. 3596	T	AY906948	MT118218
*Paragaeumannomycesrubicundus*	S.M.H. 3221	T	MT118242	MT118224
*Phaeodischloridiumaquaticum*	MFLUCC 18–1341	T	MK828639	MK835841
*Phialoarthrobotryumtriseptatum*	CBS 120.84	T	MH861706	MH873417
*Phialogeniculataguadalcanalensis*	MFLUCC 18–0260	T	MK828625	MK835825
*Phialogeniculataguadalcanalensis*	NN044662		OL627622	OL655032
*Phialosporostilbescutiformis*	MFLUCC 17–0227	T	MH758194	MH758207
*Phialosporostilbescutiformis*	MFLUCC 22–0053		ON678180	ON678145
*Phialoturbellacalva*	ICMP 23826	T	MW984585	MW984567
*Phialoturbellalunata*	MFLUCC 18–0642	T	MK828624	MK835824
*Polynemapodocarpi*	CBS 144415	T	MH327797	MH327833
*Pseudodinemasporiumfabiforme*	CBS 140010		KR611889	KR611906
*Pseudolachneafraxini*	CBS 113701	T	JQ889287	JQ889301
*Pseudolachneahispidula*	MAFF 244365		AB934072	AB934048
*Pseudolachnellaasymmetrica*	MAFF 244366	T	AB934073	AB934049
*Pseudolachnellascolecospora*	MAFF 244379		AB934086	AB934062
***Pseudolomaanthathailandica***	**MFLUCC 24–0521**	**T**	**PQ625465**	**PQ625467**
*Pseudothozetellalunata*	CGMCC 3.20661	T	OL628034	OL655157
*Psilobotrysminutus*	CBS 877.73		OR134733	OR134677
*Psilobotrysminutus*	CBS 145632		OR134734	OR134678
*Rattaniasetulifera*	GUFCC 15501	T	GU191794	HM171322
*Riisgaardialongispora*	CGMCC 3.20794	T	OL627701	OL655085
*Riisgaardiaobclavata*	CGMCC 3.20787	T	OL627568	OL655013
*Riisgaardiavermiculata*	NN042952		OL627555	OL655007
*Spadicocephalafusca*	CBS 301.85		AF486122	MH873571
*Spadicocephalafusca*	CBS 300.85		MH861882	MH873570
*Spicatisporafennica*	CBS 101641		OR134735	OR134679
*Sporendocladiabeijingensis*	CGMCC 3.20738	T	OL628290	OL655217
*Sporendocladiafumosa*	NN047731		OL627669	OL655065
*Sporoschismahemipsilum*	MUCL 56487		MW987829	MW987824
*Sporoschismamirabile*	CBS 144794		MW987830	MW987825
*Stanjehughesiahormiscioides*	S.M.H.2794		N/A	AF466060
*Stilbochaetamalaysiana*	IMI 312436	T	OL654121	OL654178
*Stilbochaetaramulosetula*	IMI 313452	T	OL654124	OL654181
*Striatosphaeriacastanea*	CBS 145352	T	MT118244	MT118229
*Striatosphaeriacodinaeophora*	M.R. 1230		AF178546	AF178546
***Submultiguttulisporamultiseptata***	**KUNCC 23–14145**	**T**	**PQ625466**	**PQ625468**
*Tainosphaeriacecropiae*	CBS 101687	T	MW984586	MW984568
*Tainosphaeriacrassiparies*	S.M.H. 1934	T	MW984587	AF466089
*Tainosphaeriellaaquatica*	MFLUCC 17–2370	T	MZ161197	MZ161195
*Tainosphaeriellathailandense*	MFLUCC 18–1282	T	MZ161198	MZ161196
*Thozetellacristata*	CBS 101112		OL654126	OL654183
*Thozetellatocklaiensis*	CBS 378.58	T	OL654128	OL654185
*Verhulstiabiformis*	NN077655		OL628434	OL655237
*Verhulstiatrisororum*	CBS 143234	T	MG022181	MG022160
*Zanclosporanovae-zelandiae*	ICMP 15781	T	MW144429	MW144411
*Zanclosporaramifera*	ICMP 22738	T	MW144433	MW144415
*Zanclosporaiberica*	CBS 130426	T	KY853480	KY853544
*Zanclosporiellaminuta*	S.M.H. 3396		N/A	AF466075

Note: status: T denotes type strains; “N/A” indicates data unavailable in GenBank. The newly generated sequences are indicated in **bold**.

The fasta files were converted to formats required for the AliView program (Larsson 2014), PHYLIP for maximum likelihood analysis (ML), and NEXUS for Bayesian inference (BI). Phylogenetic analyses were performed through the CIPRES science Gateway CIPRES science Gateway V. 3.3 (https://www.phylo.org/portal2/home.action; Miller et al. 2010). Maximum likelihood analysis was performed using RAxML-HPC v.8 tool with rapid bootstrap analysis, followed by 1000 bootstrap replicates (Miller et al. 2010; Stamatakis 2014). The final tree was selected from the suboptimal trees of each run by comparing likelihood scores under the GTRGAMMA substitution model. Bayesian analysis was performed in MrBayes 3.2.7a (Ronquist et al. 2012). The best-fit substitution model GRT + I + G was decided for all two genes by MrModeltest 2.3 under the Akaike Information Criterion (AIC) (Nylander 2004). The Markov Chain Monte Carlo (MCMC) sampling approach was used to calculate posterior probabilities (PP) (Rannala and Yang 1996; Huelsenbeck 2001; Zhaxybayeva and Gogarten 2002). Four simultaneous Markov chains were run for 1 million generations, with trees sampled every 100 generations, resulting in 10,000 trees. The first 2,000 trees, representing the burn-in phase of the analyses, were discarded, and the remaining trees were used for calculating posterior probabilities (PPs) in the majority rule consensus tree (Larget and Simon 1999).

Phylogenetic trees were visualized using FigTree v.1.4.4 (Rambaut 2014), and the layouts were reorganized using the methods described by Xie et al. (2023) and finalized with Adobe Illustrator CS6 software (Adobe Systems, USA). Sequences generated from our collections were deposited in GenBank and are listed in Table 1. Decisions regarding the discovery of new species or records were made following the guidelines of Maharachchikumbura et al. (2021).

### ﻿Phylogenetic analysis results

**Figure 1.**
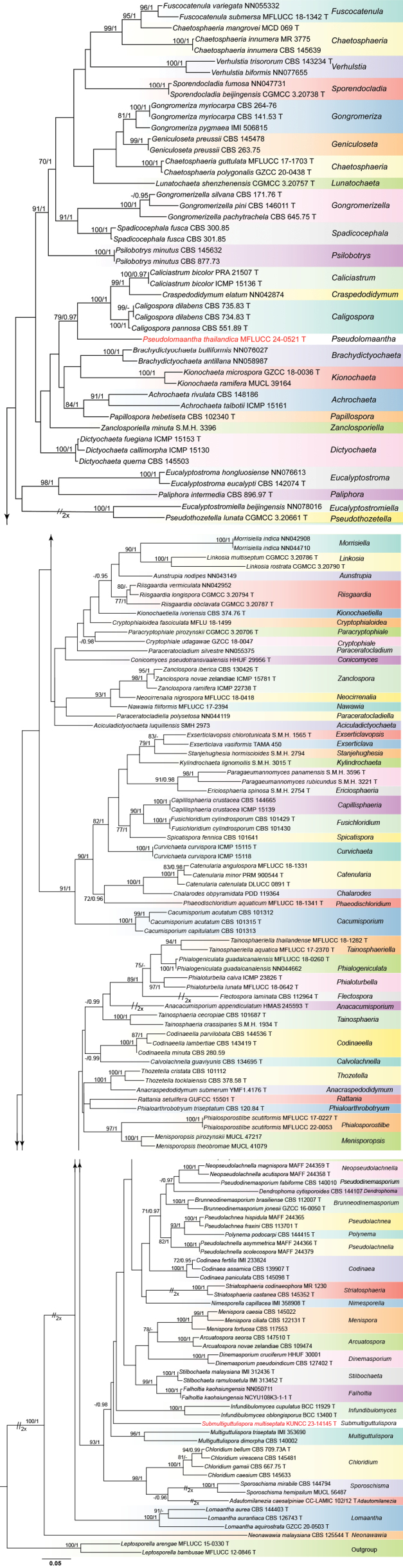
The phylogenetic tree generated from ML analysis is based on a concatenated LSU-ITS dataset for the Chaetosphaeriaceae family. Bootstrap support values for ML equal to or greater than 75% and Bayesian posterior probabilities (PPs) equal to or greater than 0.95 were indicated above or below the nodes as ML/PP. *Leptosporellaarengae* (MFLUCC 15–0330) and *L.bambusae* (MFLUCC 12–0846) were selected as the outgroup taxa. The newly obtained sequences are indicated in red.

The partial LSU-ITS nucleotide sequences were used to determine the phylogenetic position of the new taxa within the family Chaetosphaeriaceae. The concatenated sequence matrix comprises 157 ingroup taxa of Chaetosphaeriaceae and two outgroup taxa, *Leptosporellaarengae* (MFLUCC 15–0330) and *L.bambusae* (MFLUCC 12–0846). After alignment, the dataset contained 1,450 characters (LSU: 861 bp, ITS: 589 bp), including 853 distinct alignment patterns, with 11.93% comprising undetermined characters or gaps. Base frequencies were as follows: A = 0.224314, C = 0.274605, G = 0.307808, and T = 0.193272. Substitution rates were AC = 1.327038, AG = 1.998330, AT = 1.575283, CG = 0.648947, CT = 6.385392, and GT = 1.000000, with a tree length of 12.245369. The distribution shape parameter (α) was calculated as 0.317788. The ML and BI trees displayed similar topologies with no significant differences. The best-scoring RAxML tree is shown in Fig. 1, with a final likelihood value of -31034.684968.

Our two isolates were identified as *Pseudolomaanthathailandica* gen. et sp. nov. and *Submultiguttulisporamultiseptata* gen. et sp. nov. in Chaetosphaeriaceae. *Pseudolomaantha* shares a sister relationship with a clade comprising *Caliciastrum*, *Caligospora*, and *Craspedodidymum*, while *Submultiguttulispora* forms a separate clade within Chaetosphaeriaceae that is close to *Multiguttulispora*. Both genera represent distinct, independent lineages and do not belong to any existing genera within Chaetosphaeriaceae.

## ﻿Taxonomy

### ﻿ Pseudolomaantha

Taxon classificationFungiChaetosphaerialesChaetosphaeriaceae

J.Y. Zhang, Y.Z. Lu & K.D. Hyde gen. nov.

FE3D2B07-D7F0-54F0-A30E-7C0B557B6184

Index Fungorum: IF903140

Facesoffungi Number: FoF16983

#### Etymology.

The name refers to the new genus’s similarity to the genus “*Lomaantha*”.

#### Type species.

*Pseudolomaanthathailandica* J.Y. Zhang, Y.Z. Lu & K.D. Hyde

#### Description.

***Saprobic*** on dead stems of bamboo in terrestrial habitats. ***Sexual morph*** Undetermined. ***Asexual morph*** Colonies on natural substrate, effuse, scattered, hairy, dark brown, glistening. ***Mycelium*** partly immersed, composed of brown hyphae. ***Conidiophores*** macronematous, mononematous, cylindrical, straight or slightly flexuous, septate, dark brown to pale brown. ***Conidiogenous cells*** integrated, terminal, holoblastic, monoblastic, cylindrical, brown or pale brown at the apex. ***Conidia*** acrogenous, solitary, rostrate, tapering to the round apex, truncate at base, straight or slightly curved, septate, with distoseptate, pale brown to dark brown; with a gold and glistening sheath near the apex.

### ﻿ Pseudolomaantha thailandica

Taxon classificationFungiChaetosphaerialesChaetosphaeriaceae

J.Y. Zhang, Y.Z. Lu & K.D. Hyde sp. nov.

56EE1EE2-14B5-52C6-93B4-2A6E624529EA

Index Fungorum: IF903138

Facesoffungi Number: FoF16984

Fig. 2

#### Etymology.

The name refers to the country “Thailand” from where the holotype was collected.

#### Holotype.

MFLU 24-0394.

#### Description.

***Saprobic*** on dead stems of bamboo in a terrestrial habitat. ***Sexual morph*** Undetermined. ***Asexual morph*** Hyphomycetous. ***Colonies*** on natural substrate superficial, effuse, scattered, hairy, dark brown, with gold glistening on the apex of conidia. Mycelium partly immersed, partly superficial, composed of septate, mostly unbranched, smooth, brown hyphae. ***Conidiophores*** 176–275 × 6–9(–11) µm (x̄ = 219.6 × 7.5 µm, n = 20), macronematous, mononematous, solitary, cylindrical, straight or slightly flexuous, septate, black at the base, paler to light brown or brown towards the apex. ***Conidiogenous cells*** 12–22 × 5.5–7 µm (x̄ = 16.4 × 6.1 µm, n = 20), integrated, terminal, holoblastic, monoblastic, cylindrical, brown or pale brown at the apex. ***Conidia*** (92.5–)95–112.5 × 12.5–15.5 µm (x̄ = 105.8 × 13.8 µm, n = 25), acrogenous, solitary, dry, pyriform to obclavate, rostrate, tapering to the round apex, truncate at base, basal cell conical-truncate, straight or slightly curved, up to 12-septate, with distoseptate, not constricted or slightly constricted at septum, guttulate, brown, two upper cells subhyaline to hyaline, with gold and glistening appendages around the apex of the conidia.

**Figure 2.**
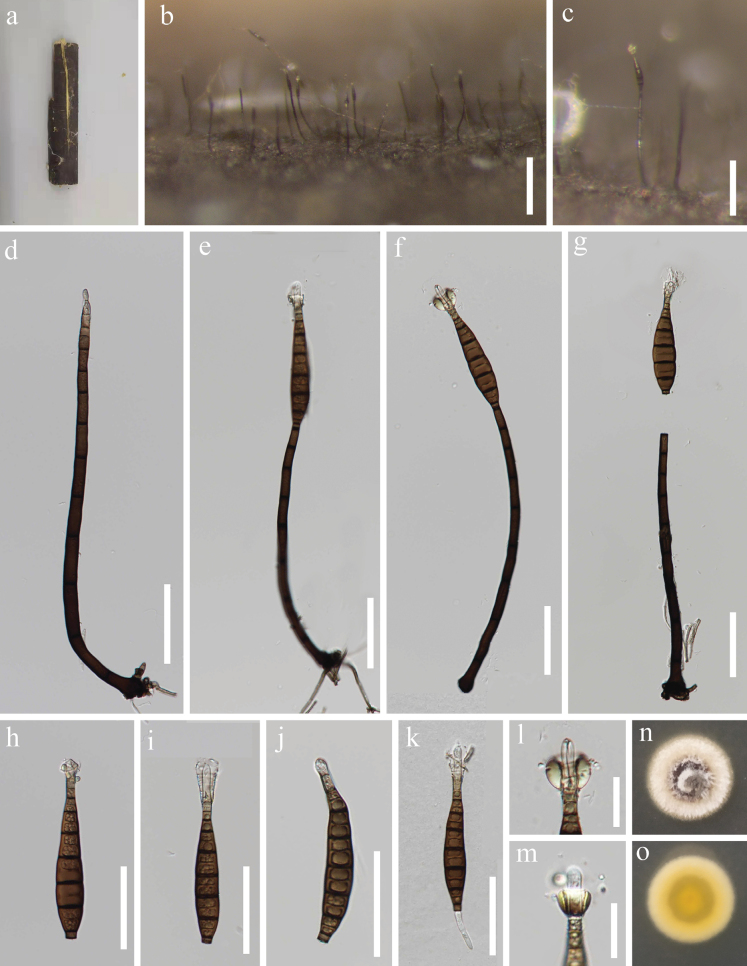
*Pseudolomaanthathailandica* (MFLU 24–0394, holotype) **a** the host substrate **b, c** colonies on the host substrate **d–g** conidiophores with conidiogenous cells **h–k** conidia **l, m** conidial appendage **n, o** pure culture from front and reverse. Scale bars: 200 µm (**b**); 100 µm (**c**); 50 µm (**d–k**); 20 µm (**l, m**).

#### Culture characteristics.

Conidia germinating on WA within 15 h and germ tube produced from the ends of conidia. Colonies growing on PDA, reaching 22–26 mm in 20 days at 26 °C, circular, edge entire, umbonate with a knobby protuberance, white from above; zonate, yellowish orange in the center, grayish olive to yellowish towards to margin from below.

#### Material examined.

Thailand • Chiangmai Province, Mushroom Research Center (MRC), on dead stems of bamboo, 11 September 2020, H.W. Shen, Y205-1 (MFLU 24–0394, ***holotype***), ex-type living culture, MFLUCC 24–0521.

#### Notes.

BLAST results for the ITS and LSU sequence data of *Pseudolomaanthathailandica* show 88.39% similarities with *Caligosporadilabens* (CBS 735.83) and 97.81% similarities with *Craspedodidymumelatum* (NN042874), respectively. Phylogenetic analysis shows that *Pseudolomaanthathailandica* forms a distinct lineage basal to *Caliciastrum*, *Caligospora*, and *Craspedodidymum* with statistical support (79% ML/0.97 PP, Fig. 1). Members of *Caliciastrum*, *Caligospora*, and *Craspedodidymum* are characterized by phialidic conidiogenous cells with open, vase-shaped collarettes, and brown or hyaline conidia. In contrast, our new species has a sporidesmium-like asexual morph with non-phialidic conidiogenous cells (Figueroa et al. 2018; Wu and Diao 2022; Réblová and Nekvindová 2023). Morphologically, *Pseudolomaantha* resembles *Lomaantha* in having macronematous, mononematous conidiophores, integrated holoblastic conidiogenous cells, and acrogenous, obclavate, rostrate, distoseptate, pale brown to brown conidia (Wu and Zhuang 2005; Wu and Diao 2022; Réblová and Nekvindová 2023). However, the two genera are phylogenetically distinct. Additionally, *Lomaantha* species have conidiogenous cells that are determinate or extend percurrently a few times, as well as conidia that lack or bear filiform, extended, simple or branched apical appendages and distinct septal pores (Wu and Zhuang 2005; Wu and Diao 2022; Réblová and Nekvindová 2023). In contrast, *Pseudolomaantha* has determinate conidiogenous cells, conidia with golden, glistening appendages at the conidial apex, and lack distinct pores in the distosepta. Based on the combination of morphological and phylogenetic evidence, *Pseudolomaantha* is introduced as a new genus to accommodate *P.thailandica* within Chaetosphaeriaceae.

### ﻿ Submultiguttulispora

Taxon classificationFungiChaetosphaerialesChaetosphaeriaceae

J.Y. Zhang, Y.Z. Lu & K.D. Hyde gen. nov.

5B3B2579-A7C9-5351-9F53-A2DE2E3C8964

Index Fungorum: IF903141

Facesoffungi Number: FoF16985

#### Etymology.

The name refers to the new genus’s close affinity with the genus “*Multiguttulispora*”.

#### Type species.

*Submultiguttulisporamultiseptatum* J.Y. Zhang, Y.Z. Lu & K.D. Hyde.

#### Description.

***Saprobic*** on dead wood. ***Sexual morph*** Undetermined. ***Asexual morph Colonies*** on natural substrate, effuse, single, or gregarious, brown to black. ***Mycelium*** partly immersed, composed of brown hyphae. ***Conidiophores*** macronematous, mononematous, single or in small groups, septate, dark brown at the base becoming light brown towards the apex. ***Conidiogenous cells*** integrated, mono- to polyphialidic, terminal to lateral, with funnel-shaped collarettes, cylindrical to cylindrical-lageniform, brown to pale brown to subhyaline towards the apex. ***Conidia*** acropleurogenous, septate, pale brown to olive green to brown, with subhyaline cells at both ends of the conidia, fusiform, or ellipsoidal-fusiform, with a filiform appendage at each end.

### ﻿ Submultiguttulispora multiseptata

Taxon classificationFungiChaetosphaerialesChaetosphaeriaceae

J.Y. Zhang, K.D. Hyde & Y.Z. Lu sp. nov.

B30C6EE3-0BDC-50E5-9426-327E23573FCC

Index Fungorum: IF903139

Facesoffungi Number: FoF16986

Fig. 3

#### Etymology.

The name refers to the multi-septate conidia of the new species.

#### Holotype.

HKAS 129868.

#### Description.

***Saprobic*** on a dead wood log by a stream. ***Sexual morph*** undetermined. ***Asexual morph*** Hyphomycetous. ***Colonies*** on natural substrate superficial, effuse, single, or gregarious, arise in groups from knots of hyphal cells, brown to black. ***Mycelium*** partly superficial, partly immersed, composed of septate, pale brown to brown, smooth-walled hyphae. ***Conidiophores*** 285–385(–533) µm long × 5–7 µm wide at the base (x̄ = 341 × 6 µm, n = 15), macronematous, mononematous, single or clustered in groups, erect, straight or flexible, unbranched, septate, smooth, guttulate, dark brown or black at the base, becoming pale brown towards the apex. ***Conidiogenous cells*** 64.5–100 × 4.3–6.1 µm (x̄ = 80.2 × 5.2 µm, n = 15), mono- to polyphialidic, with discrete, terminal to lateral phialides, integrated, terminal, with lateral openings formed by successive sympodial elongation, cylindrical to cylindrical–lageniform, with funnel-shaped collarettes, smooth-walled, guttulate, brown at the base and becoming pale brown to subhyaline towards the apex. ***Conidia*** 33–40 × 7.5–9 µm (x̄ = 36.6 × 8.3 µm, n = 20), acropleurogenous, 5(–6)-septate, not constricted at the septum, pale brown to olive green to brown, with subhyaline cells at both ends, straight, sometimes slightly curved, occasionally guttulate, fusiform, or ellipsoidal-fusiform, with a filiform, short and hyaline appendage at each end.

**Figure 3.**
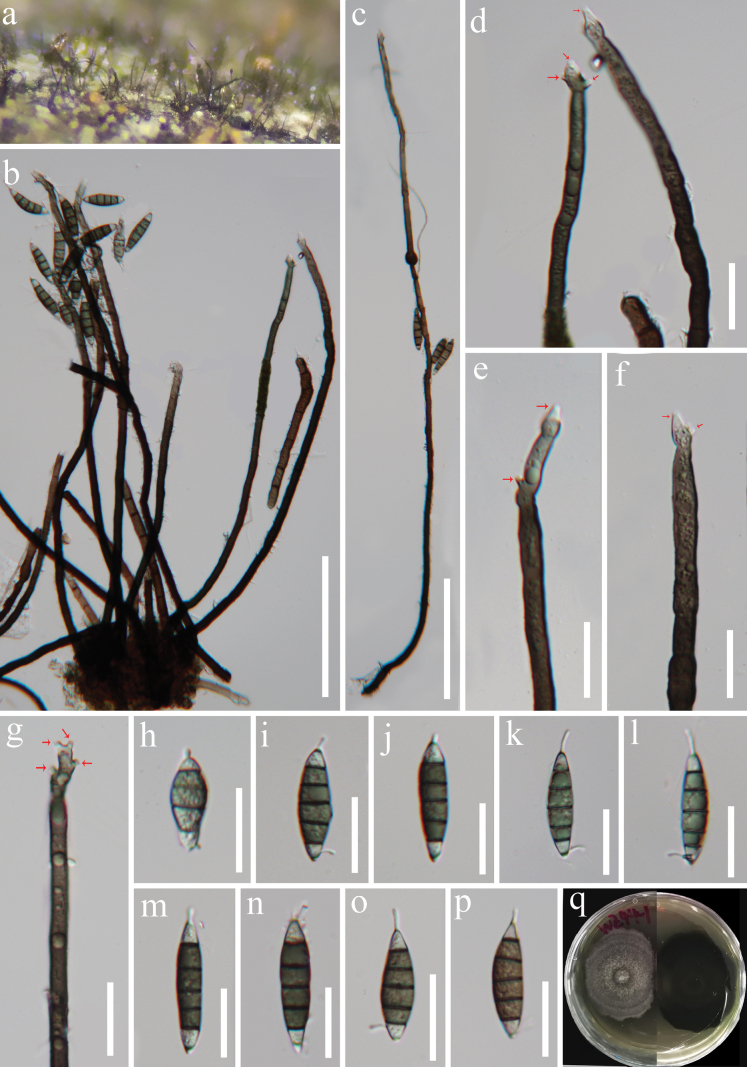
*Submultiguttulisporamultiseptata* (HKAS 129868, holotype) **a** colonies on the host substratum **b, c** conidiophores **d–g** conidiogenous cells (arrows showing conidiogenous loci) **h–p** conidia **q** pure culture from front and reverse. Scale bars: 100 µm (**b, c**); 20 µm (**d–p**).

#### Culture characteristics.

Conidia germinating on WA within 15 h and germ tube produced from conidia. Colonies growing on PDA, reaching 35–40 mm diameter in 15 days at 26 °C, circular with slightly irregular edge, flat with a protuberance in the center, dry, velvety, zonate, tephrosiousto to grey from center to margin; dark brown or black from below.

#### Material examined.

China • Hainan Province, Wuzhishan City, Wuzhishan Tropical Rainforest Scenic Area, on a dead wood log by a stream, 15 August 2021, J.Y. Zhang, WZ44-1 (HKAS 129868, holotype; GZAAS 23–0763, isotype); ex-type living cultures, KUNCC 23–14145.

#### Notes.

Based on a BLASTn search in GenBank, the ITS and LSU sequences of our new collection show 91.92% and 95.35% similarity to *Phialogeniculataguadalcanalensis* (NN044662) and *Multiguttulisporatriseptata* (IMI 353690), respectively. The phylogenetic tree indicates that our new isolate forms a distinct lineage closely related to *Multiguttulispora*, without statistical support. This lack of support may be attributed to the absence of molecular sequences of many close phylogenetic relatives, which remain undiscovered (Hyde et al. 2024c). *Submultiguttulispora* shares similarities with *Multiguttulispora* in the absence of setae and the presence of macronematous conidiophores with polyphialidic conidiogenous cells that exhibit sympodial extension. Both genera produce septate conidia with a filiform, hyaline appendage at each end. However, *Submultiguttulispora* is distinguished from *Multiguttulispora* by its fusiform or ellipsoidal-fusiform, dematiaceous conidia, whereas the conidia of *Multiguttulispora* are cylindrical, oblong, and hyaline. Based on these morphological and phylogenetic differences, a new genus, *Submultiguttulispora*, is introduced to accommodate our new isolate, *S.multiseptata*.

## ﻿Discussion

In this study, *Pseudolomaanthathailandica* gen. et sp. nov. and *Submultiguttulisporamultiseptata* gen. et sp. nov. were introduced based on morphological characteristics and phylogenetic analyses. These two species exhibit non-phialidic and phialidic asexual morphs, respectively. The introduction of these new taxa further highlights the richness and diversity of anamorphic chaetosphaeriaceous fungi (Réblová et al. 2021a, b, c, d, e; Wu and Diao 2022).

The characteristics of conidiophores, conidiogenous cells, and conidia are particularly important for delimiting asexual genera in Chaetosphaeriaceae, along with the presence or absence of appendages (Réblová et al. 1999, 2021b, c, d; Lin et al. 2019; Zheng et al. 2020; Wu and Diao 2022). A significant number of anamorphic chaetosphaeriaceous genera produce hyaline or subhyaline conidia in various shapes, often with filiform, hyaline setulae at the ends, as seen in genera like *Arcuatospora*, *Codinaea*, and *Kinochaeta* (Hughes and Kendrick 1968; Réblová et al. 2020, 2021b, c; Wu and Diao 2022; Hyde et al. 2024b). In contrast, many hyphomycetous genera with dematiaceous conidia lack setulae, such as *Catenularia*, *Phaeodischloridium* and *Sporoschisma* (Goh et al. 1997; Yang et al. 2016; Réblová et al. 2021e; Wu and Diao 2022). *Submultiguttulisporamultiseptata* gen. et sp. nov. resembles other anamorphic chaetosphaeriaceous genera in having phialidic conidiogenous cells and conidia with filiform, hyaline setulae at both ends (Réblová and Gams 2000; Liu et al. 2016; Lin et al. 2019; Wu and Diao 2022). However, it is distinct in its well-developed conidiophores, polyphialidic conidiogenous cells, and pale brown to olive green to brown, septate conidia with hyaline setulae at each end. The latest key to phialidic asexual genera in Chaetosphaeriaceae was provided by Wu and Diao (2022).

Wu and Diao (2022) recognized ten hyphomycetous genera with non-phialidic anamorphs in Chaetosphaeriaceae, *viz.*, *Aunstrupia*, *Ellisembia*, *Falholtia*, *Linkosia*, *Lomaantha*, *Morrisiella*, *Paliphora*, *Riisgaardia*, *Stanjehughesia*, and *Zanclospora*. Subsequently, a new non-phialidic genus, *Neocirrenalia*, characterized by dark brown or black helicoid conidia, was added to this family (Meyers and Moore 1960; Somrithipol et al. 2002; Yang et al. 2023). Recently, Delgado et al. (2024) reclassified *Ellisembia* into Sporidesmiaceae (Sordariomycetes) based on analyses of a newly collected type species, *E.coronata*, and expanded and emended *Lomaantha* to include related ellisembia-like taxa within a monophyletic lineage in Chaetosphaeriaceae. Currently, Chaetosphaeriaceae comprises 10 non-phialidic hyphomycetous genera. Most of these genera are sporidesmium-like, with exceptions such as *Neocirrenalia* (a helicosporous genus) and *Paliphora*, which is characterized by setiform conidiophores, polytretic conidiogenous cells, and subfusiform to subacerose, hyaline conidia (Gusmão et al. 2008; Shenoy et al. 2010; Goh et al. 2014; Malosso et al. 2017; Wu and Diao 2022; Yang et al. 2023; Ma et al. 2024). In this study, we introduced a new genus, *Pseudolomaantha*, which also exhibits a sporidesmium-like asexual morph, characterized by well-developed, solitary or clustered conidiophores and pyriform to obclavate conidia with a glistening gold appendage around the apex, but is phylogenetically distinct. A key to hyphomycetous genera with non-phialidic anamorphs is provided herein.

### ﻿Key to hyphomycetous genera with non-phialidic anamorphs

**Table:** 

1	Sporidesmium-like genera	**2**
–	Not sporidesmium-like genera	**3**
2	Conidiophores absent (reduced to conidiogenous cells), or solitary or in a small group	**4**
–	Conidiophores in synnemata	**5**
3	Conidiogenous cells polytretic; conidia hyaline, subfusiform to subacerose	***Paliphora***
–	Conidiogenous cells monoblastic; conidia black, helicoid	***Neocirrenalia***
4	Conidia with appendage at the apex	**6**
–	Conidia without appendage	**7**
5	Conidia euseptate	***Falholtia***
–	Conidia distoseptate	***Morrisiella***
6	Conidiophores absent or well-developed, conidia cylindrical, obclavate or narrowly fusiform, often with filamentous, hyaline apical appendages and typically bearing distinct pores in the distosepta	***Lomaantha***
–	Conidiophores well-developed, conidia pyriform to obclavate, with a gold and glistening appendage around the apex, and distinct pores are not observed	***Pseudolomaantha***
7	Synanamorph of *Zanclospora* with phialides	***Zanclospora***
–	Not synanamorph of *Zanclospora*	**8**
8	Conidiophores absent; conidia euseptate	**9**
–	Conidiophores absent, conidia distoseptate	***Linkosia***
9	Conidia obclavate, obclavate-rostrate, subcylindrical	***Riisgaardia***
–	Conidia cylindrical, clavate, or obclavate	***Stanjehughesia***

## Supplementary Material

XML Treatment for Pseudolomaantha

XML Treatment for Pseudolomaantha thailandica

XML Treatment for Submultiguttulispora

XML Treatment for Submultiguttulispora multiseptata

## Data Availability

All of the data that support the findings of this study are available in the main text.

## ﻿Additional information

### Author ORCIDs

Jing-Yi Zhang https://orcid.org/0000-0003-0606-6169

Kevin D. Hyde https://orcid.org/0000-0002-2191-0762

Jian Ma https://orcid.org/0009-0008-1291-640X

Na Wu https://orcid.org/0000-0002-4837-9019

Fatimah Al-Otibi https://orcid.org/0000-0003-3629-5755

Li-Juan Zhang https://orcid.org/0000-0002-3234-6757

Yong-Zhong Lu https://orcid.org/0000-0002-1033-5782
